# Preparation and Characterization of Multilayered Microcapsules of *Lacticaseibacillus rhamnosus* Encapsulated With Sodium Alginate, Jujube Mucilage, and Whey Protein Isolate in Goat Milk Dessert

**DOI:** 10.1002/fsn3.71450

**Published:** 2026-02-12

**Authors:** Sara Baleshzar, Seyed Saeed Sekhavatizadeh

**Affiliations:** ^1^ Department of Microbiology, School of Basic Sciences, Kazerun Branch Islamic Azad University Kazerun Iran; ^2^ Food Science and Technology Department Fars Agricultural and Natural Resources Research and Education Center, AREEO Shiraz Fars Iran

**Keywords:** acid and salt condition, gasterointestinal condition, heat stress, SEM, texture analysis

## Abstract

This research aimed to investigate the ability of a two‐layer extrusion encapsulation technique to protect *Lacticaseibacillus rhamnosus* (LR) during simulated gastrointestinal conditions (SGC) and heat stress. The microcapsules were made with alginate as the first layer, whereas the second layer consisted of jujube mucilage and whey protein isolate (WPI). The encapsulation efficiency (EE%), microstructure, and survival rate (SR%) under heat stress were assessed to determine the best formulation for addition to a goat milk dessert (GMD). The number of LRs during storage, microstructure, acidity, and texture were evaluated during storage. The microencapsulated LR (MLR) received the highest EE% rating. It contained JM and WPI at a ratio of 6:4 (94.16%) (JW3). JW3 had the best SR (69.14%) after 15 min of heat stress. The GMD containing MLR‐JW3 (GMD‐MLR‐JW3) demonstrated a superior SR in terms of storage (75.48%) and SGC (70.98%) than did the GMD containing free LR (GMD‐FLR). The incorporation of MLR‐JW3 led to increases in acidity (20.98°D), hardness (47.25 ± 1.25 g), cohesiveness (0.51 ± 0.02), gumminess (24.15 ± 0.35 g), and chewiness (1.4 ± 0.04 mJ), but adhesiveness and springiness were nearly constant among the GMD samples. Scanning electron microscopy demonstrated that the addition of beads led to a decrease in the quantity of pores and casein aggregates, culminating in a denser microstructure within the GMD‐MLR. This research highlights the effective implementation of a dual‐layer microencapsulation system aimed at improving LR survival and functionality in GMDs, thereby providing enhanced probiotic stability and texture for the advancement of functional food development.

## Graphical abstract

A dual‐layer microencapsulation system composed of alginate, jujube mucilage, and whey protein isolate effectively protected *Lacticaseibacillus rhamnosus* under heat and simulated gastrointestinal conditions. The optimized formulation (JM:WPI, 6:4) achieved the highest encapsulation efficiency (94.16%). Incorporation into goat milk dessert improved probiotic stability, acidity, hardness, and overall texture, highlighting its potential for functional dairy product enhancement.

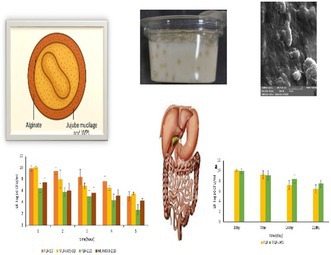

## 1 Introduction

The International Scientific Association for Probiotics and Prebiotics (ISAPP) defines probiotics as “live microorganisms which, when delivered in sufficient amounts, elicit a beneficial effect on host health.” According to this definition, for probiotics to truly make a difference in terms of treatment and prevention, they need to be included in the final product at a certain concentration—specifically, at least 6 log CFU/g or milliliter (Taşkoparan et al. 2025). Lactobacillus and Bifidobacterium are two key players in the gut microbiota and are often used as probiotics. *Lacticaseibacillus rhamnosus* (LR) stands out among the Lactobacillus strains. This strain is well known as a functional probiotic and has been extensively studied and added to fermented dairy products because of its impressive probiotic benefits, including enhancing immunity, maintaining oral health, lowering cholesterol levels, regulating intestinal mucosal health, improving lipid metabolism, alleviating resistance to diabetes, and allergic reactions (Feng et al. 2025). It is used in naturally fermented milk products, ice cream, yogurt, and cheese (Gao et al. 2021).

Probiotics have shown some great health benefits, but a few key conditions need to be realized. For example, keeping probiotics stable and ensuring that they remain alive during food production or storage can be quite a challenge for the industry (Suez et al. 2019). In addition to enduring the stresses of manufacturing, storage, and transportation, probiotic products must retain their activity and viability as they pass through the gastrointestinal tract (D'Amico et al. 2025). This could lead to a decrease in the number of living cells, which would significantly reduce their effectiveness. To address this problem, microencapsulation technology is used to wrap bacteria in protective materials, forming a stable barrier that shields probiotics from environmental stress. Among the various encapsulation techniques, the layer‐by‐layer encapsulation method demonstrates considerable potential in safeguarding probiotics from adverse environmental conditions while maintaining cell viability. The layer‐by‐layer (LbL) assemblies are capable of significantly mitigating environmental harm to the encapsulated cells, thereby preserving their metabolic activity and functionality (Virk et al. 2025).

The materials chosen for encapsulation need to meet specific criteria, such as being safe and nontoxic, having excellent film‐forming properties, being highly soluble in the intestine, and being biocompatible with probiotics to ensure that they remain viable (Rao et al. 2025).

One of the polymers is sodium alginate. This polymer has great biocompatibility, cost‐effectiveness, and easy availability, making it an ideal choice for wall materials that encapsulate bioactive substances, especially in food products. Alginate, which is an anionic hydrophilic polysaccharide, consists of D‐mannuronic and L‐guluronic monosaccharides linked together by 1,4‐β‐glycosidic bonds (Barbosa‐Nuñez et al. 2025). Sodium alginate hydrogels have several notable disadvantages, such as structural instability and a lack of sufficient mechanical strength, which can limit their practical application (Koosha et al. 2025). In addition, many studies indicate that alginate microcapsules demonstrate porosity and stability only within the pH range of 6–9 (Lalarukh et al. 2025). To fill sodium alginate pores, a composite material with a second layer is proposed. Carbohydrates and proteins may be used as composite materials.

Whey protein isolate (WPI) offers significant nutritional benefits. It is distinguished by its vital functional attributes, such as emulsification, gelling, and ability to create films. Given these properties and its comparatively low price, WPI is a great option for encapsulating hydrophobic substances (Mazzocchi et al. 2025). Utilizing WPI within an encapsulation or delivery system guarantees the protection of the encapsulated materials, facilitating targeted delivery to the intestine. Researchers have found that the inherent properties of WPI play a crucial role in the successful encapsulation of probiotics and other compounds. For instance, WPI forms composites with maltodextrin and gum Arabic to improve the storage stability of bioactive substances, including mandarin essential oils (Virk et al. 2025).

Hydrocolloids are derived from various sources. One of them is jujube ( *Ziziphus jujuba* Mill.) mucilage, which has not been applied as a second layer in extrusion encapsulation. Jujube is widely acknowledged as the most significant species among the Zizyphus species for fruit production in the buckthorn family, Rhamnacea (Pu et al. 2017). The JM can be considered a particular type of complex polysaccharide. These compounds are mostly branched by L‐galacturonic acid, D‐xylose, D‐glucose, arabinose, and L‐rhamnose. The application of pseudoplastic hydrocolloids such as JM can significantly improve the viscosity and stability of food materials (Pu et al. 2017). The microencapsulated wall material may include carbohydrates and proteins, modified or natural, used in extrusion. These materials can protect probiotics from the harsh conditions in the SGC (Homayouni‐Rad et al. 2024).

In the literature review, no studies have investigated encapsulated LR with alginate, JM, and WPI by extrusion in GMD. The main aim of the current study was to increase LAB survival during storage and the SGC. Our research focused predominantly on LR survival under harsh conditions. Moreover, the physicochemical properties of free and encapsulated LR‐supplemented GMD were assessed during storage.

## 2 Materials and Methods

### 2.1 Materials

Freeze‐dried LR ATCC 53103 was obtained from the Persian Type Culture Collection, Tehran, Iran. Peptone water, MRS broth, sodium citrate, and MRS agar were obtained from Merck (Merck, Darmstadt, Germany). Sodium alginate was acquired from Sigma (Sigma, Steinheim, Germany). Jujube seeds were obtained from a local market in Shiraz, Iran. WPI was prepared from Behin Azma, Fars, Iran.

### 2.2 Extraction of Mucilage From Jujube Powder

First, the contaminants present on the jujube fruits were removed. The fruity pulp material was subsequently isolated, compressed, and blended via a blender (Moulinex Perfect MIX^+^ LM88HD27, France). The samples were maintained at 4°C before the mucilage extraction process. In summary, the procedure involved the addition of 200 mL of distilled water to 2.0 g of jujube pulp powder. The mixture was heated at 100°C and rotated for 20 min at 400 rpm with a magnetic stirrer (Shimifan, Iran). Fine cotton cloth was subsequently used to separate the waste materials. The process was conducted with a Büchner funnel and centrifugation (Labtron, Iran) at 3800×*g* for 15 min. The mucilage mixture, also referred to as the supernatant mixture, was obtained and subsequently precipitated with ethanol (with a ratio of 1:4). Following this process, the mixture was maintained at 4°C for 24 h. After that, the mucilage was filtered with a cotton cloth and then dried at 27°C for 48 h (Yekta and Ansari 2019).

### 2.3 Bacterial Preparation

Lyophilized LR was cultivated in MRS broth under anaerobic conditions at 30°C for 48 h. The culture media were subsequently centrifuged at 2264×*g* at 4°C for 10 min. The culture media underwent a triple washing process with sterile saline before being suspended in 0.1% w/v peptone water.

### 2.4 *Lacticaseibacillus rhamnosus* Encapsulation Procedures

After the LR cultures were activated, they were separately centrifuged at 2800×*g* for 10 min. Each LR suspension had a volume of 5 mL, with an approximate bacterial concentration of 9.99 log CFU/mL in MRS broth prior to centrifugation. Microencapsulation was carried out via the extrusion technique. For this method, 5 mL of the centrifuged LR culture was mixed with 15 mL of a 1.5% (w/v) sodium alginate solution. The mixture was then slowly dripped through a 0.11 mm needle into a sterile 0.1 M CaCl_2_ solution to form beads. After allowing the beads to set for 12 h, they were washed with 0.1% (w/v) peptone water. Next, the beads were gently stirred for 40 min in either JM or WPI coating solutions, as specified in Table S1, under low rotating conditions (1 × g). Following several rinsing steps, the microencapsulated LR (MLR) beads were finally rinsed with sterilized 0.1% (w/v) peptone water. To assess bead morphology, images were taken via an Olympus BX51 optical microscope (Japan), and measurements were made via microscope software (version 1.07). The aspect ratio of the beads was calculated via the formula described in Equation (1).

(1) $$\text{Aspect ratio}=\frac{\text{Major Axis}\;\left(\mathrm{mm}\right)}{\text{Minor Axis}\;\left(\mathrm{mm}\right)\;}$$

### 2.5 SEM

The lyophilized samples were carefully placed onto an aluminum holder and then coated with gold via a Desk Sputter Coater DSR1 from Nanostructural Coating Co. in Iran, all of which were set for examination under a scanning electron microscope (SEM, VEGA3, TESCAN, Czech Republic). The analysis was conducted at a voltage of 10.0 kV, ensuring that the distance between the microscope lens and the sample surface was kept within a range of 8.91–7.03 mm throughout the process.

### 2.6 Encapsulation Efficiency

The encapsulation efficiency (EE) was calculated via Equation (2):

(2) $$\mathrm{EE}\%=\frac{\log\;N}{\log\;N_{0}}\times 100$$

In this formula, “*N*” represents the number of LR obtained after encapsulation (log CFU/g microspheres), and “N0” represents the number of LR initially applied to prepare the beads (log CFU/g exists in mixed alginate).

### 2.7 Color

To assess color characteristics, a colorimeter (Konica Minolta CR400, Japan) was employed for both the MLR samples and the GMD formulations. Prior to measurement, the instrument was calibrated using the standard reference values of L* = 97.63, a* = 0.32, and b* = 1.93. For analysis, approximately 5 g of each sample (MLR or GMD) was placed into the designated measurement chamber, where parameters related to brightness (L*), redness (a*), and yellowness (b*) were recorded and evaluated.

### 2.8 Heat Resistance of the FLR and MLRs

All FLR and MLRs were suspended in MRS broth and subsequently incubated in a water bath maintained at 72°C. At predetermined time intervals (0, 5, 10, and 15 min), samples were collected, serially diluted, and plated onto MRS agar for anaerobic cultivation to assess bacterial viability.

### 2.9 Survival Under Acidic and Salt Conditions

A 15% w/v sodium chloride solution mixed with glycine‐HCl buffer at a pH of 1.5 was used to assess the survival rates of the MLRs and FLR. We collected samples at 1, 2, 3, and 4 h intervals, followed by a series of dilutions. To culture the LRs, we employed the pour plate technique and then incubated the plates at 37°C for 2 days.

### 2.10 GMD Production

Skimmed goat milk powder was dissolved in a 1.0 M NaCl solution to obtain a final concentration of 10% (w/w). This blend was then stirred at 10 × g via a mechanical stirrer. Next, we added modified starch and sucrose to the milk base and stirred the mixture continuously for 30 min at a steady temperature of 27°C. Specifically, κ‐carrageenan was dispersed in a 0.1 M NaCl solution and agitated at 20 × g for 30 min at the same temperature. This solution was then pasteurized at 75°C for 10 min. The final formulation GMD contained, per 100 g of product, 2 g of modified starch, 0.1 g of κ‐carrageenan, and 8 g of sucrose. All the components were combined and mixed for an additional 5 min at 50°C. In this study, three identical batches of GMD were prepared. FLR and MLR‐JW3 cultures were inoculated into two of the batches with approximately equal numbers of bacteria, while one batch was left untreated as the control (C).

All the samples were heated to 80°C and then quickly plunged into cold water for 1 min before being stored at 4°C. During storage, various microbiological and physicochemical properties of the GMD were monitored, including the survival of MLR and FLR, color parameters, titratable acidity, texture, and pH.

### 2.11 Acidity and pH of GMD

The titratable acidity of the GMD was determined via the Durnic titration method. A calibrated pH meter (Greisinger Electronic, Germany) was used to measure the pH (Sahan et al. 2008).

### 2.12 Texture of the GMD

Texture profile analysis (TPA) of the GMD was conducted on the 1st and 21st days of storage via a Brookfield CT3 texture analyzer (Model 4500, USA) equipped with a TA11/1000 cylindrical probe. The test involved compressing GMD samples to 40% of their original height via a two‐cycle compression method. Cubic samples with dimensions of 30 mm in height and 20 mm in diameter were used, with the penetration depth set at 20 mm. The testing speed was maintained at 10 mm/s during the pre‐test and post‐test phases and at 1 mm/s during the actual test. The following textural parameters were evaluated: hardness (g), cohesiveness, chewiness (mJ), adhesiveness (mJ), springiness, and gumminess (g). All the measurements were performed at a controlled ambient temperature of 25°C ± 3°C.

### 2.13 LR Survival in GMD

For the selective cultivation and enumeration of LRs, a novel selective medium, modified rhamnose 2,3,5‐triphenyl tetrazolium chloride–LBS–vancomycin agar (M‐RTLV agar), was used. Before plating, 25 g of each GMD sample was diluted with sterile trisodium citrate solution (2% w/v). Microbial culture and enumeration were carried out on Days 1, 7, 14, and 21 to monitor the survival and viability of LRs during storage.

### 2.14 Survival of FLR and MLR Exposed to SGC

The survival of the MLR and FLR under SGC was assessed. One gram of each dessert sample containing either FLR or MLR, collected on Days 1 and 21 postproduction, was separately suspended in normal saline. We adjusted the pH of the suspensions to between 1.4 and 1.9 by using 1 N HCl. Subsequently, pepsin (from porcine stomach mucosa) and lipase extracted from *Rhizopus oryzae* (from Sigma Aldrich, Dorset, UK) were added to achieve final concentrations of 3 g/L and 0.9 mg/L, respectively. After that, the samples were placed in a shaker incubator and incubated at 37°C (Labtron, Tehran, Iran) at approximately 110 rpm for 2 h to simulate the gastric phase. Following the gastric phase, the pH of the samples was adjusted to 4.3–5.2 by adding 150 mL of 1 N NaOH to a solution containing 14 g of NaH_2_PO_4_·2H_2_O and distilled water up to 1 L. Bovine bile and pancreatin, which are derived from porcine pancreas (Sigma Aldrich), were added to reach final concentrations of 10 and 1 g/L, respectively. This was done to simulate the initial intestinal environment, known as enteric phase 1. After that, the samples were incubated at 37°C under agitation for an additional 2 h. In the final step, the pH was increased to 6.7–7.5, and the bile and pancreatin concentrations were maintained at 10 and 1 g/L, respectively, to mimic the later stage of the intestinal environment (enteric phase 2). The samples were incubated under the same conditions for another 2 h, resulting in a total of 6 h of simulated digestion.

Probiotic counts (LRs) were determined at 30 min, 2, 4, and 6 h postinitiation of the assay. For each time point, triplicate samples were taken from separate flasks prepared under identical conditions. Appropriate volumes (ranging from 0.01 to 1 mL) of each sample were pour‐plated onto M‐RTLV agar. The plates were incubated at 37°C for 72 h under anaerobic conditions. Colony counts were used to assess survival rates, which were calculated according to Equation (3).

(3) $$\text{Survival rate}\;\left(\%\right)=\frac{\;N\;\left(\text{logCFu}/\mathrm{mL}\right)\;}{N0\;\left(\log\;\mathrm{CFU}/\mathrm{mL}\right)}\times 100$$

*N*: the number of viable LRs in log CFU/mL after exposure to SGF, and *N*_0_: the number of viable LRs in log CFU/mL before exposure to SGC.

### 2.15 Statistical Analysis

The data were analyzed via SPSS software, version 22. To ensure that the results were reliable, all the experimental procedures were carried out in triplicate. We used one‐way analysis of variance (ANOVA) to statistically assess the data over the storage period and then applied Duncan's multiple range test to pinpoint significant differences between treatment means at the 5% significance level. Additionally, we created graphical representations of the data via Microsoft Excel 2019.

## 3 Results and Discussion

### 3.1 Size, Aspect Ratio, and Encapsulation Efficiency (EE%) of the Beads

**FIGURE 1:**
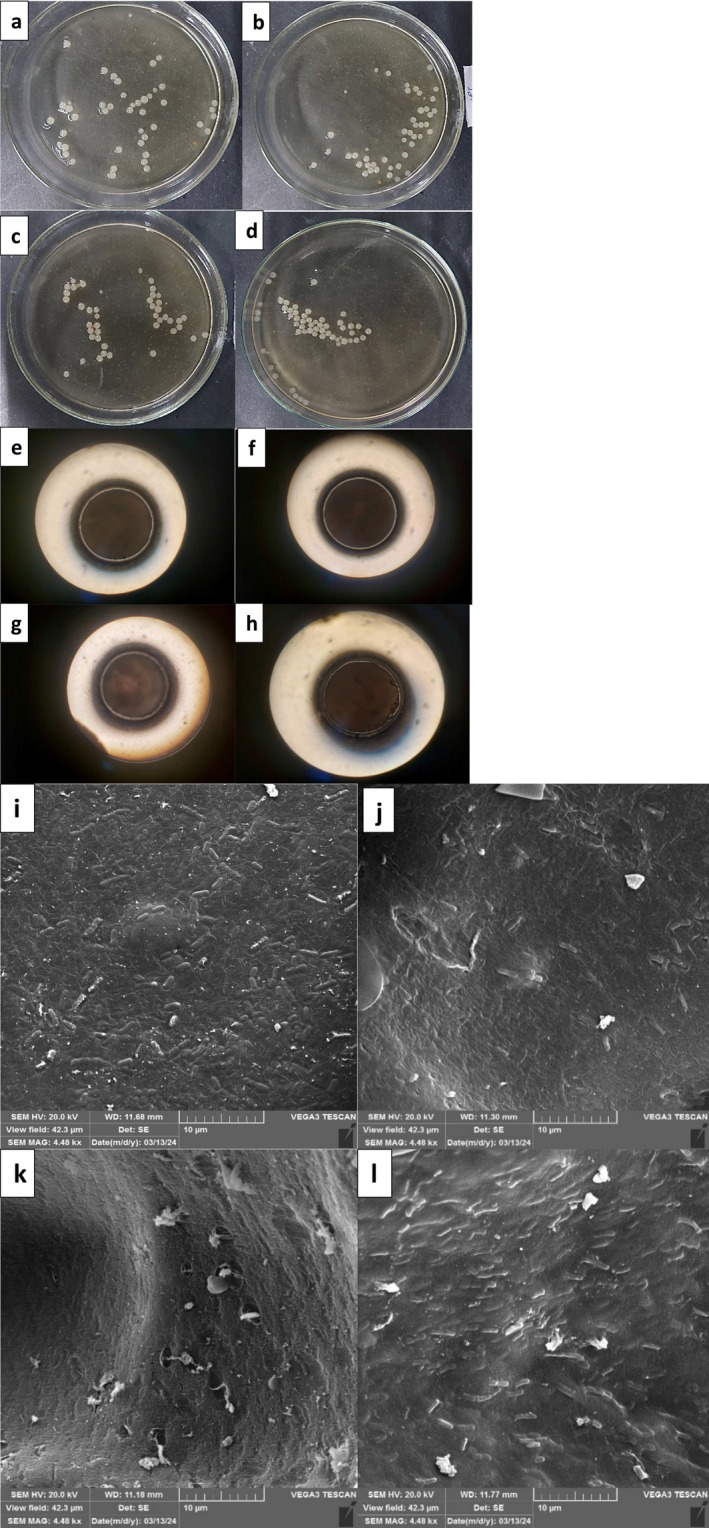
Photography image of MLR at concentrations of (a) 0.8% and 0.2%, (b) 0.6% and 0.4%, (c) 0.4% and 0.6%, (d) 0.2% and 0.8% of WPI and JM, respectively. Light microscopy of MLR at concentrations of (e) 0.8% and 0.2%, (f) 0.6% and 0.4%, (g) 0.4% and 0.6%, (h) 0.2% and 0.8% of WPI and JM, respectively (40×) and Scan electron microscopy of MLR at concentrations of (i) 0.8% and 0.2%, (j) 0.6% and 0.4%, (k) 0.4% and 0.6%, (l) 0.2% and 0.8% of WPI and JM, respectively. JM, jujube mucilage; LR, *Lacticaseibacillus rhamnosus*; MLR, microencapsulated *Lacticaseibacillus rhamnosus*; WPI, Whey protein isolate.

The alginate layers of various types of beads were identical in diameter (Table 1). The second layer of beads produced with WPI and JM had mean diameters ranging from 19.61 ± 0.51 to 23.33 ± 0.69 μm. The maximum second‐layer bead diameter belonged to JW4 (23.33 μm). The data showed that increasing the JM concentration led to an increase in the second layer size. Dehkordi et al. (2020) reported that the mean diameter of alginate–WPI microcapsules ranged from 33 to 180 μm, depending on polymer concentrations and alginate/WPI ratios. Formulations containing WPI exhibited lower viscosity and more uniform, smaller particles compared to pure alginate systems, although higher overall polymer concentrations could increase particle size. Moreover, WPI contributes to a reduction in microcapsule size due to the formation of a more compact and cohesive matrix, whereas mucilage tends to increase particle size because of its high viscosity and long‐chain polysaccharide structure (Yang et al. 2025). The dimension of the coating layers in probiotic beads is highly important because of its critical role in maintaining bacterial viability throughout storage. Furthermore, in addition to serving as a barrier that regulates the access of water and nutrients to probiotic bacteria, the coating also protects bacteria against harsh environmental conditions (Agriopoulou et al. 2024). All the beads were spherical in shape (Figure 1). The aspect ratios were between 1.09 and 1.12. Castro‐Rosas et al. (2021) reported comparable results when they produced alginate microspheres using extrusion. The sphericity of these microspheres is affected by multiple factors, such as the molecular weight of alginate, intrinsic viscosity, agitation speed, degree of polymerization, alginate concentration, and the air pressure applied. There's a direct correlation between higher agitation and pressure, which increases the chances of the beads breaking. The shape of calcium alginate beads plays a crucial role in their mechanical and chemical stability. For example, research has indicated that beads that are not perfectly spherical tend to have lower gel strength. The breakage and cracking have been noted in tear‐shaped and irregular beads, which can lead to unwanted release of the material inside. Additionally, beads that are uniform in size and have a clear shape help ensure consistent reactions and reliable controlled‐release profiles (Lee et al. 2013).

**TABLE 1:** Layers dimension and color parameters in microencapsulated Lacticaseibacillus rhamnosus.

Bead		Treatments			
Parameters		JW1	JW2	JW3	JW4
Layers dimention (μm)	Alginate Layer	3779.78 ± 16.67^a^	3699.44 ± 56.70 ^a^	3815.0 ± 49.43^a^	3670.67 ± 53.66^a^
	Second Layer	19.61 ± 0.51^b^	19.89 ± 1.56^b^	22.44 ± 0.91^ab^	23.33 ± 0.69^a^
Color parameters	L*	65.67 ± 2.19^a^	65.78 ± 2.25^a^	60.56 ± 1.02^ab^	58.78 ± 1.27^b^
	a*	−0.44 ± 0.18^b^	−0.67 ± 0.24^b^	0.22 ± 0.22^a^	0.56 ± 0.18^a^
	b*	5.22 ± 0.40^c^	6.78 ± 0.32^b^	7.89 ± 0.63^ab^	8.44 ± 0.34^a^
EE%		85.95 ± 4.67^a^	89.65 ± 5.0^a^	94.16 ± 7.72^a^	92.94 ± 5.47^a^

*Note:* Data (mean ± standard error) are from three replications (*n* = 3). Means followed by different lowercase in row indicate significant different (*P* ≤ 0.05) by Duncan test. JW1 (0.8 and 0.2); JW2 (0.6 and 0.4); JW3 (0.4 and 0.6); JW4 (0.2 and 0.8) percent of WPI and JM respectively used in encapsulation.

Abbreviations: EE%, encapsulation efficiency; JM, jujube mucilage; LR, *Lacticaseibacillus rhamnosus*; WPI, whey protein isolate.

We also evaluated the encapsulation efficiency of LR in beads prepared with JM and WPI alone and in combination. As shown in Table 1, the LR encapsulation efficiency of the beads was maximized in JW3 (94.16% ± 7.72%) at a JM:WPI ratio of 4:6. The high encapsulation efficiency (EE%). We suggests that the multilayer design effectively captures viable cells within the beads. This increase in EE% for the protein–polysaccharide combination is likely due to the equal proportions of these components in the secondary bead matrix, which leads to the creation of stronger and more rigid structures than those made with single polymers. Polysaccharides and protein materials can be blended together to encapsulate probiotics. For instance, Xu et al. (2023) utilized a combination of tea protein and xanthan gum to encapsulate probiotics, which greatly enhanced their ability to survive in harsh conditions.

We can see further evidence of this in the electron microscopy results (Figure 1i–l). The enhanced structural integrity is probably a result of hydrophobic interactions within the protein–polysaccharide complex, which help create a more stable and resilient matrix (Naseem et al. 2025). This improvement in encapsulation efficiency may also be attributed to the strong hydrogen bonding and electrostatic interactions between JM and WPI. These interactions contribute to the formation of a tighter, more cohesive matrix, which enhances the entrapment of the encapsulated material. The encapsulation efficiency achieved in this study is comparable to the findings of Eratte et al. (2015), who reported that protein–polysaccharide complex coacervates composed of gum arabic and whey protein isolate were more effective at encapsulating omega‐3 fatty acids and probiotic bacteria than single‐component wall materials.

Upon mixing 5 mL of centrifuged LR with 15 mL of sodium alginate solution (1.5% w/v) and subsequently dripping this mixture into a 0.1 M calcium chloride solution under standard cross‐linking conditions, the resulting wet calcium alginate beads had an average mass of 15.10 g. The viable bacterial count at this stage was 10.29 log_10_ CFU/g. Following the secondary coating step with JM and WPI, the average mass of the coated microcapsules (wet weight) was approximately 15.31 g and the viable bacterial count after coating remained high, averaging 9.63 log_10_ CFU/g.

### 3.2 SEM of Beads

To assess the morphological traits of the MLR, SEM was used. Our results match previous studies that noted how cracks can facilitate the escape of encapsulated components, which can affect the stability of beads (Youssef et al. 2021). The shape and features of the beads can change depending on the type of coating material used as a wall material in encapsulation. As a result, the cells that are encapsulated tend to be scattered randomly throughout the biopolymer matrix (Figure 1i–l). Mixtures with more WPI (e.g., 0.8% WPI + 0.2% JM) form tight, smooth layers owing to strong protein bonds, but these layers can trap bacteria due to their low porosity. In hydrocolloids with a significant amount of whey protein isolate, WPI molecules can be solvated in water through the formation of intermolecular hydrogen bonds (H‐bonds) at specific sites on the protein surface. These H‐bonds can cause the protein structure to change, which exposes hydrophobic groups within WPIs. This ultimately promotes protein aggregation (Fan et al. 2022). The results of this study align with earlier research, which suggested that cracks can facilitate the diffusion of encapsulated components, ultimately increasing the stability of the beads at risk. Typically, the morphological characteristics of beads vary on the basis of the specific type of wall material employed in the formation of a dual biopolymer (Youssef et al. 2021). On the other hand, mucilage creates spongy, water‐rich networks (Tantiwatcharothai and Prachayawarakorn 2020). This kind of wall material may keep bacteria alive, but they struggle to maintain their shape. The way proteins and carbohydrates combine to form conjugates enhances the encapsulation properties by changing the physical traits of the wall. This finding shows how adjusting the mixture of these materials can fine‐tune microcapsules for better probiotic delivery.

### 3.3 Color of the Beads

A colorimetric analysis was performed on the beads to examine the effects of JM and WPI on their color development (Table 1). In the bead wall formulation, we found that increasing the JM content and decreasing the WPI concentration led to a decrease in the L*, but an increase in a*, and b* color parameters. This shows that the transparency of the layers of beads decreased, but the yellowness and redness increased with increasing content of JM. The JM and WPI are transparent with yellow, red, and white colors, respectively. These materials can cover the LR. This was confirmed by the SEM image (Figure 1k). In a study, the addition of jujube mucilage to yogurt resulted in a significant decrease in the L* value, while the a* and b* values increased compared to the control. These alterations in yogurt color may be attributed to interactions between jujube polysaccharides and milk proteins, such as whey and casein (Yekta and Ansari 2019).

### 3.4 Heat Resistance of the FLR and MLRs

**FIGURE 2:**
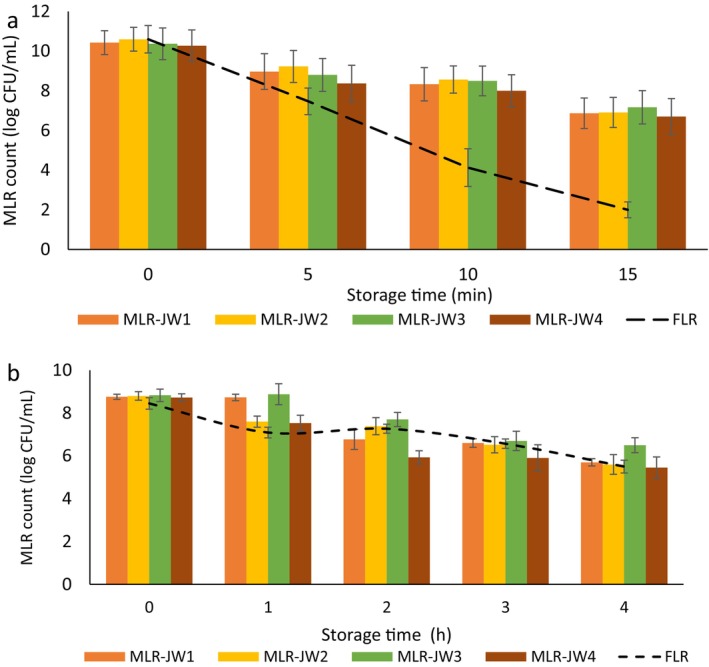
The survival of free *Lacticaseibacillus rhamnosus* (FLR) and microencapsulated LR (MLR) at concentrations of MLR‐JW1 (0.8% and 0.2%); MLR‐JW2 (0.6% and 0.4%); MLR‐JW3 (0.4% and 0.6%); MLR‐JW4 (0.2% and 0.8%) percent of WPI and JM respectively at 72°C (a), acidic (pH 1.5) and salt (15% NaCl) conditions (b). JM, jujube mucilage; LR, *Lacticaseibacillus rhamnosus*; WPI, Whey protein isolate. Data (mean ± standard error) are from three replications.

As shown in Figure 2a, when FLR cells were exposed to moist heat at 70°C for 15 min, they were eliminated (2.0 log CFU/mL). On the other hand, the viability of the MLR cells encapsulated in the JM‐WPI‐coated microcapsules only slightly decreased to 3.2 log CFU/g in JW3 because of their impressive ability to protect the cells during heat exposure. The materials used for the walls may also improve the density of the bead matrix network (Wongsasulak et al. 2007). In related studies, at the maximum experimental temperature of 70°C, *L. sakei* J15 demonstrated the highest survival rate of 31.95% when subjected to a whey protein isolate (WPI): sodium hyaluronate (SH) ratio of 1:3 in bead wall material formulation. In contrast, LR S8 showed a survival rate of 20.55% at a WPI:SH ratio of 3:1. Overall, the encapsulation of probiotic cells utilizing the WPI‐SH complex exhibited enhanced protective effects at elevated temperatures. The heat sensitivity inherent in Lactobacilli significantly diminishes their functionality, as thermal exposure compromises the integrity of the cell membrane (Zhou et al. 2024).

In this study, we found that when JM and WPI molecules were exposed to moist heat, they came together to form a gel, which could increase the density of the matrix network. Additionally, the SEM images (Figure 1k) show that JM and WPI effectively coat the surfaces of the bacteria. The combination of JW3 strengthens the gel structure of the bead wall matrix, resulting in a better barrier against moisture and heat penetration in the microcapsules (Wongsasulak et al. 2006). We hypothesize that JM and WPI can soak up moisture from the composite matrix, which helps stop hot moisture from seeping into the microcapsules.

### 3.5 Survival Under Acidic and Salt Conditions

The effects of 15% NaCl in a highly acidic environment (pH 1.5) on the viability of MLRs (JW1–JW4) and FLR are illustrated in Figure 2b. These findings indicate that increasing the incubation time in saline + acidic conditions resulted in a decrease in the growth of MLRs and FLR.

According to the findings of Ghadermazi et al. (2019) and Igartúa et al. (2022), the electrostatic complexation between WPI and anionic polysaccharides such as quince seed mucilage (QSM) or soluble soybean polysaccharides (SSPS) really enhances resistance to acidic and saline conditions. In acidic environments (with a pH of about 3.5–4.0), WPI has a net positive charge, while QSM or SSPS remains negatively charged due to deprotonated carboxyl groups. This interaction creates coacervates or nanocomplexes that protect WPI from aggregating near its isoelectric point. This protective layer reduces the contact between proteins and stabilizes the system. When NaCl is added (up to 100 mM), the polysaccharide layer provides both steric and electrostatic repulsion, which helps counteract charge screening and maintain colloidal stability. Therefore, this biopolymer complex acts as an effective protective matrix, making it perfect for encapsulating bioactives in acidic or ion‐rich food systems.

This reduction in water activity, combined with the increased osmotic pressure from higher intracellular sodium levels, might play a role in the lower viability of the cell population. Additionally, a statistically significant difference (*p* ≤ 0.05) in survival rates was noted between the MLRs and FLR. After 4 h of exposure to these conditions, MLR‐JW3 presented the highest survival rate, at 73.61%, outperforming the other samples. At this 4‐h point, only MLR‐JW3 maintained the threshold level of LR in food (6 log CFU/mL), while the other samples did not survive. Electron microscopy results revealed that the JW3 sample had the fewest surface cavities. This reduced number of cavities on the outer layer may help acid and salt penetrate the bead more effectively. As a result, this could increase the survival of LRs in both saline and acidic environments. Additionally, the results from this experiment align well with the outcomes of the heat resistance test conducted in this study.

Our results align with those of Teoh et al. (2011), who reported that the growth rate of free *L. acidophilus* LA1 cells was not as significant as that of encapsulated cells when subjected to higher sodium concentrations and longer incubation times. These findings suggest that encapsulation could increase the survival of *L. acidophilus* under salty and acidic conditions. Additionally, the outcomes of this experiment are in line with the heat resistance tests conducted in this study (Teoh et al. 2011).

In a study by De Castro‐Cislaghi et al. (2012), it was reported that in whey without added salt, the viable cell counts for both free and microencapsulated Bifidobacterium Bb‐12 remained relatively close, with values between 10.07 ± 0.06 log CFU/mL and 10.02 ± 0.07 log CFU/g (De Castro‐Cislaghi et al. 2012). This finding is not consistent with the results of the present study. Additionally, previous studies have demonstrated that chitosan and sodium alginate are excellent materials for encapsulating LR, highlighting their important role in protecting these bacteria under acidic conditions (Athayde et al. 2024). We hypothesize that the electrostatic interaction between WPI and JM under acidic conditions was strongest because of their high opposite charges, which resulted in the most compact complexation. The net charges of JM and WPI were negative and positive, respectively. In a similar study, WPI carried a net positive charge and a negative charge on chitosan at low pH, which resulted in the most compact complexation (Gunasekaran et al. 2007; Xu et al. 2021).

### 3.6 pH and Titratable Acidity of GMD

**FIGURE 3:**
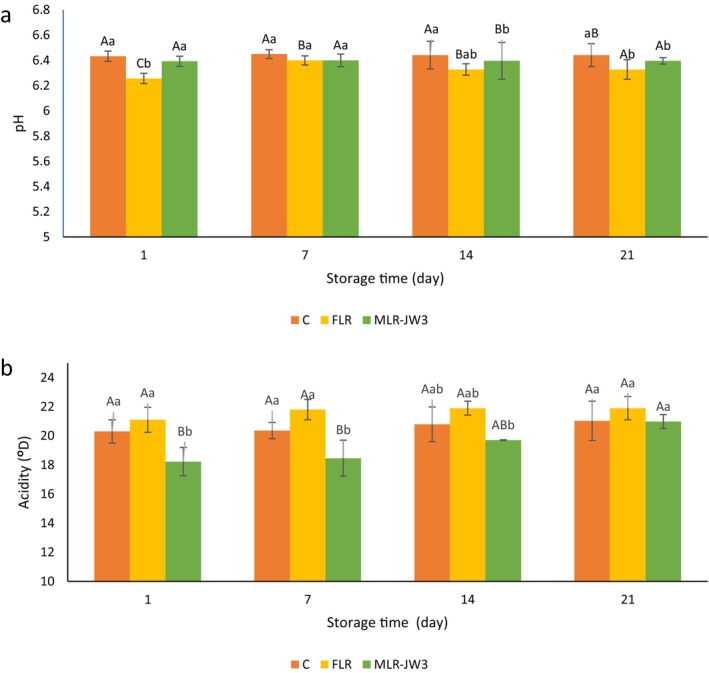
pH (a) and acidity (b) values of free *Lacticaseibacillus rhamnosus* (FLR) and microencapsulated *Lacticaseibacillus rhamnosus* (MLR) and control dessert (C) during storage time. Data (mean ± standard error) are from three replications. Means followed by different uppercase letters indicate significant differences (*p* ≤ 0.05) among different days within the same sample, as determined by Duncan's multiple range test, whereas means followed by different lowercase letters indicate significant differences (*p* ≤ 0.05) among different samples at the same time point, as determined by the same test.

The pH and titratable acidity results are presented in Figure 3. Although the pH remained nearly constant among the samples, the titratable acidity increased during storage. This phenomenon may be attributed to the fact that pH and acidity are not always inversely correlated, as the buffering capacity of the matrix and the dissociation constants of the organic acids can influence the measured pH values. Indeed, Paulson and Stevens (1974) reported that pH and titratable acidity may vary independently, particularly in food systems. The findings of this study are not consistent with those reported by Azarkhavarani et al. (2019). In their investigation, *Enterobacter faecium* was incorporated into a beverage. The results demonstrated that postacidification was notably lower in beverage samples containing free and encapsulated bacteria. A potential explanation for this discrepancy may lie in the bacterial strain utilized in Azarkhavarani et al. (2019) study, as *E. faecium* exhibited minimal postacidification at 4°C. The acidity of FLR had the greatest value among the samples at the end of storage, but the acid production rate in MLR‐JW3 was 0.14/day.

During the storage period, the FLR dessert exhibited negligible changes in acidity, whereas the MLR‐JW3 dessert showed a significant increase in acidity by the final week of storage. During the first 3 weeks of storage, the acidity of the MLR‐JW3 sample remained stable, likely due to encapsulation, which restricted the cells' access to nutrients and thereby limited fermentative acid production (Cence et al. 2025). As the storage time progressed, the acidity of the MLR‐JW3 sample rose, likely due to the bead wall weakening and the texture developing larger cavities. Although FLR produced some acidic metabolites, the acidity of the GMD dessert remained nearly unchanged throughout the 21‐day storage period, suggesting that most free probiotic cells had lost their metabolic activity, likely due to unfavorable conditions such as low pH, limited nutrient availability, and exposure to oxygen or heat, a common limitation of non‐encapsulated probiotics in dairy matrices. This observation is consistent with findings from da Silva Simões et al. (2025), who reported similar results when using edible alginate–fungal chitosan coatings as carriers for *L. casei* in strawberries.

### 3.7 Texture of the GMD

The textural qualities of the GMDs, including hardness, springiness, adhesiveness, cohesiveness, gumminess, and chewiness, were analyzed via TPA testing (Table 2). Hardness is a key feature of food, as it reflects the amount of force needed to compress a substance between your tongue and the roof of your mouth (Fox et al. 2017). As shown in Table 2, the hardness decreased during storage time in all the samples. The addition of beads containing JM + WPI significantly increased the hardness of the GMD (47.25 ± 1.25 g) among the samples. In agreement with the findings of this study, Bušić et al. (2018) and Lee et al. (2013) reported that adding filler materials to alginate solutions significantly improved the hardness of microgels (Bušić et al. 2018; Li et al. 2023). In a different study, Qi et al. (2020) reported that adding pectin and κ‐carrageenan made alginate‐based microgels less difficult (Qi et al. 2020).

**TABLE 2:** Color and texture parameters of GMD during 21 day of storage at 4°C.

Parameters		Day	MLR‐JW3	FLR	C
Color parameters	L*	1	56.33 ± 0.83^cC^	66.77 ± 1.11^aA^	63.55 ± 0.74^bC^
		7	65.44 ± 1.29^aA^	66.33 ± 0.64^aA^	67.0 ± 0.71^aB^
		14	61.44 ± 10.02^aB^	61.77 ± 1.07^aB^	62.66 ± 0.74^aC^
		21	68.55 ± 1.19^aA^	67.33 ± 1.40^aA^	70.0 ± 0.44^aA^
	a*	1	−0.5 ± 0.17^aA^	−0.66 ± 0.16^aA^	−0.66 ± 0.40^aB^
		7	−0.66 ± 0.16^aA^	−0.55 ± 0.17^aA^	−0.44 ± 0.24^aB^
		14	−0.55 ± 0.29^aA^	−0.33 ± 0.23^abA^	−1.33 ± 0.33^aB^
		21	−0.66 ± 0.23^aA^	−2.11 ± 0.20^cB^	−2.22 ± 0.14^aA^
	b*	1	7.11 ± 0.45^bC^	9.11 ± 0.20^aB^	9.0 ± 0.92^aB^
		7	8.88 ± 0.48^bBC^	6.77 ± 0.57^aC^	9.88 ± 0.58^aB^
		14	10.11 ± 0.20^aB^	7.33 ± 0.33^bBC^	9.55 ± 0.34^aB^
		21	12.33 ± 1.25^bA^	19.44 ± 1.05^aA^	12.11 ± 0.77^bA^
Texture parameters	Hardness (g)	1	67.55 ± 2.05^a^	55.55 ± 2.35^b^	42.60 ± 1.60^cd^
		21	47.25 ± 1.25^c^	41.50 ± 0.5^cd^	36.75 ± 1.75^d^
	Adhesiveness (mJ)	1	0.56 ± 0.09^a^	0.65 ± 0.06^a^	0.56 ± 0.04^a^
		21	0.61 ± 0.10^a^	0.87 ± 0.28^a^	0.63 ± 0.02^a^
	Cohesiveness	1	0.29 ± 0.02^c^	0.31 ± 0.03^c^	0.29 ± 0.02^c^
		21	0.51 ± 0.02^a^	0.46 ± 0.04^ab^	0.37 ± 0.04^bc^
	Springiness (mm)	1	7.99 ± 0.15^b^	8.59 ± 0.36^ab^	8.87 ± 0.12^ab^
		21	9.12 ± 0.42^ab^	9.43 ± 0.15^a^	8.70 ± 0.54^a^
	Gumminess (g)	1	20.18 ± 0.35^c^	23.0 ± 0.8^b^	11.55 ± 0.45^d^
		21	24.15 ± 0.35^ab^	25.10 ± 0.40^a^	12.25 ± 0.65^d^
	Chewiness (mJ)	1	1.57 ± 0.05^ab^	1.70 ± 0.08^a^	0.89 ± 0.05^c^
		21	1.4 ± 0.04^b^	1.55 ± 0.05^ab^	0.86 ± 0.07^c^

*Note:* Data (mean ± standard error) are from nine replications in color parameters and three replications in texture parameters. ^A–C^ Means in the same column with different uppercase letters (in color parameters) and ^a–c^ lower case letter in texture parameters differ significantly (*p* ≤ 0.05). ^a–c^ Means in the same row with different lowercase letters differ significantly (*p* ≤ 0.05). Microencapsulated LR (*Lacticaseibacillus rhamnosus*) with formulation JW3 (MLR‐JW3) containing a 60:40 ratio of jujube mucilage (JM): whey protein isolate (WPI); Free *Lacticaseibacillus rhamnosus* (FLR), goat milk dessert (GMD); L* is the luminance or lightness component, a* (from green to red) and b* (from blue to yellow).

Adhesiveness is all about the “force needed to pull away the material that clings to the mouth, usually the palate, while we're eating” (Alkhalaileh 2025). The adhesiveness was constant among the samples and during storage. The findings of Mani‐López et al. (2017) are not consistent with the results of this study. One possible reason could be the difference in the flavored alginate used in their research, which may have contained substances that supported *L. fermentum* cell adhesion and allowed the formation of a sticky gel with adhesiveness similar to that of the gelatin gel (Mani‐López et al. 2017). In contrast, in the present study, differences in the materials used and the double‐layer structure of the microencapsulated bacteria might have potentially hindered the increase in adhesion capacity.

Cohesiveness, often referred to as consistency, is all about how strong the internal connections are that make up the core of a product (Basiri et al. 2018). The cohesiveness increased during the storage time for all the samples. These findings were similar to those of previous studies (Mousavi et al. 2019). MLR‐JW3 had the most valuable number among the samples. In a similar study, *L. acidophilus* microcapsules with whey protein and xanthan as wall materials were produced. These beads are then added to yogurt. The results revealed that cohesiveness increased in supplemented yogurt (Khorshidi et al. 2021). The results of the mentioned study corroborate the findings of the current research.

Elasticity, commonly known as springiness, describes how quickly a material returns to its original shape after being deformed once the external force is removed. Interestingly, a significant negative relationship was found between hardness and springiness (Basiri et al. 2018). On the other hand, a nonsignificant difference was observed among the samples. Storage time did not have any effect on springiness, in contrast to the results of the current study by Qaziyani et al. (2019), which reported higher springiness in samples containing beads. The addition of microcapsules to the chewing gum mixture made it springier and more enjoyable to chew (Qaziyani et al. 2019). The presence of lecithin and inulin in the bead wall material is responsible for the increased springiness observed in the supplemented samples, which differs from the findings of our study. In the study by Qaziyani et al. (2019), the presence of lecithin and inulin in the microcapsule wall matrix may be responsible for the increased springiness observed in the enriched samples, which contrasts with the findings of the present study.

Gumminess is the product of firmness and cohesiveness. The gumminess showed different values that varied on the basis of the measurements taken for both hardness and cohesiveness (Dantas et al. 2021). The gumminess increased during storage. The maximum gumminess value was observed in MLR‐JW3. One possible reason for the greater gumminess observed in MLR‐JW3 may be attributed to the higher hardness index of this product. In various studies, researchers encapsulated *Lactiplantibacillus plantarum* via a mixture of sodium caseinate, inulin, and soy protein isolates. The findings revealed that encapsulated bacteria had greater gumminess than did the control sample (Virk et al. 2024). This result aligns with our results.

Chewiness refers to how many times a person needs to chew a piece of food to make it soft enough to swallow comfortably (Qaziyani et al. 2019). The chewiness was constant during storage, but it increased in the supplemented samples. Gumminess can influence chewiness. Consequently, the gumminess measurement led to a notable variation in chewiness when comparing MLR‐JW3 to other samples. This observation was made over a storage period of 21 days. These findings indicated that JM+ WPI positively influenced gumminess, chewiness, and hardness.

### 3.8 Color Parameters of the GMD

The color parameters, that is, L*, a*, and b*, of the double‐layer beads are shown in Table 2. Throughout the storage period, we observed that the L* (lightness) and b* (yellowness) values for all GMD samples increased, while a* (redness) remained fairly stable except for C‐GMD. This points to a gradual increase in brightness and yellowness over time. The typical white appearance of dairy matrices is mainly due to light scattering by colloidal particles like fat globules and casein micelles. In this study, the presence of a protein‐mucilage complex in the outer layer of the microbeads likely changed the light‐scattering properties, which in turn affected the color parameters. Notably, we found a statistically significant increase (*p* ≤ 0.05) in a* and b* for MLR‐JW3‐GMD on Day 21 of storage. This finding somewhat aligns with Bulut et al. (2022), who noted that adding chickpea protein to set yogurt raised a* and lowered L* parameters. The observed variations in the a* trends may be attributed to the specific types of protein and polysaccharide employed in the formulation, namely WPI and JM, both of which possess inherent color‐related properties that can influence the overall chromatic characteristics of the system. As shown in Table 1, adding MLR‐JW3 resulted in higher a* and b* values, suggesting that the makeup of the encapsulating biopolymer system plays a key role in shaping the visual appeal of GMDs.

In conclusion, both our study and that of Farahnaky et al. (2016) demonstrate how formulation variables directly influence the color characteristics of food‐based delivery systems. While maltodextrin tends to lighten the appearance, natural biopolymers such as JM can introduce subtle yet perceptible color shifts, offering opportunities for tailoring visual properties without synthetic additives. These insights are valuable for optimizing the sensory and aesthetic qualities of encapsulated functional foods and nutraceuticals (Farahnaky et al. 2016).

### 3.9 SEM of GMD

**FIGURE 4:**
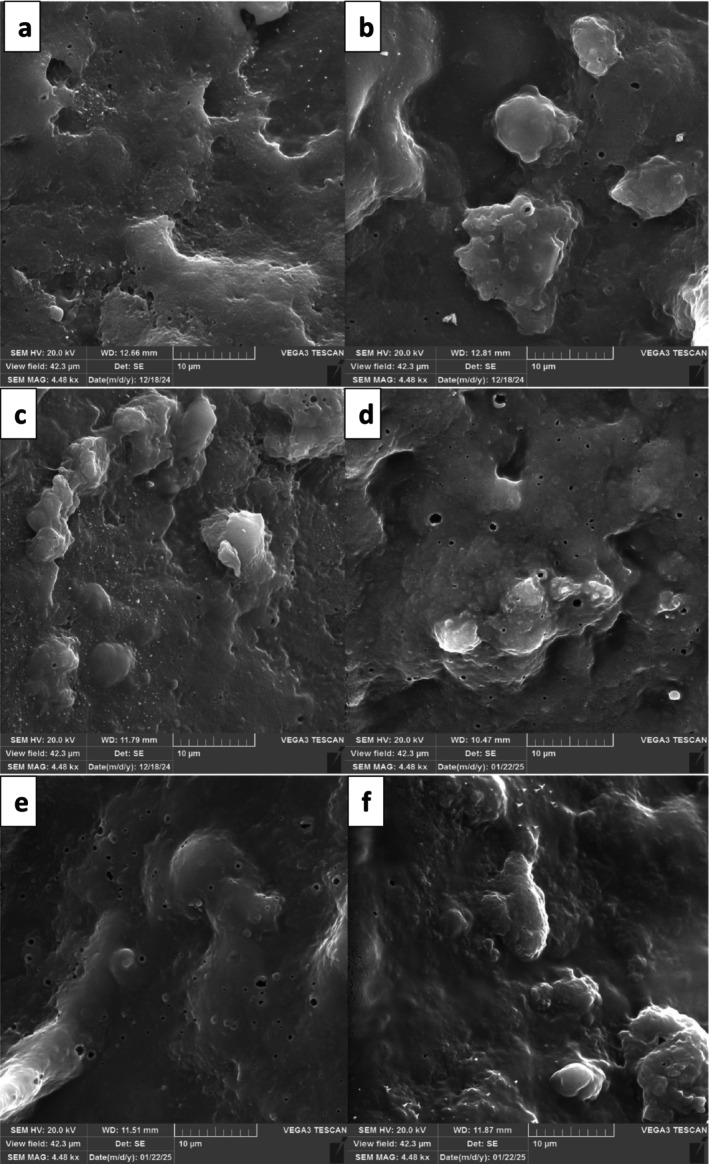
The SEM image of GMD contains microencapsulated *Lacticaseibacillus rhamnosus* (MLR), free *Lacticaseibacillus rhamnosus* (FLR), and Control (C), goat milk dessert (GMD) during storage time. C was in 1st (a); FLR in 1st (b); MLR in 1st (c); C in 21st (d); FLR in 21th (e); MLR in 21th (f) of storage time.

The microstructure of GMDs that contain probiotic bacteria, whether they are encapsulated or not, was examined by scanning electron microscopy, as shown in Figure 4a–f. The SEM images revealed a noticeable level of porosity, which is marked by holes or voids. This porosity might be due to air becoming trapped during the processing stage or could result from the drying method used to prepare the samples for SEM analysis. These voids are surrounded by a matrix that consists mainly of milk proteins (Majzoobi et al. 2016). The addition of LR and MLR‐JW3 contributed to a more compact and uniform microstructure in the goat milk dessert. This effect was particularly pronounced in the MLR‐JW3 samples after 21 days of storage, as a noticeable reduction in the number of pores and voids was observed. In a study carried out by Ghadermazi et al. (2019) on WPI and QSM, it was shown that their complex coacervation driven by strong electrostatic interactions between the positively charged WPI and the negatively charged QSM resulted in the formation of tightly packed macro‐complexes. This research indicated that microencapsulation with WPI and QSM creates a more compact and resilient structural matrix, enhancing the stability and protective capabilities of the resulting microcapsules. So, the denser structure could be attributed to protein–polysaccharide interactions between WPI, JM, and alginate, which enhance the integrity of the gel matrix. The addition of MLR‐JW3 and LR resulted in a more compact structure, which was particularly noticeable in MLR‐JW3 after 21 days of storage, as it resulted in a reduction in the number of pores and voids. Both MLR‐JW3 and FLR were effectively wrapped up and protected by the surrounding matrices. Moreover, the choice of materials for encapsulation plays a crucial role in shaping the internal structure of GMDs. The surfaces of the GMD‐MLR‐JW3 samples were smoother than those of the other formulations. Li et al. (2021) made similar observations, noting that adding beads can reduce the number of pores and cavities, which helps create a denser microstructure. This change in structure likely reduced protein rearrangement, dehydration and shrinkage during the yogurt‐making process. The modified microstructure also highlights the differences in texture among the samples. In particular, the enhanced uniformity of casein aggregation promoted more efficient gelation, ultimately resulting in increased hardness of the MLR‐JW3‐GMD sample (Table 2).

### 3.10 LR Survival in GMD

**FIGURE 5:**
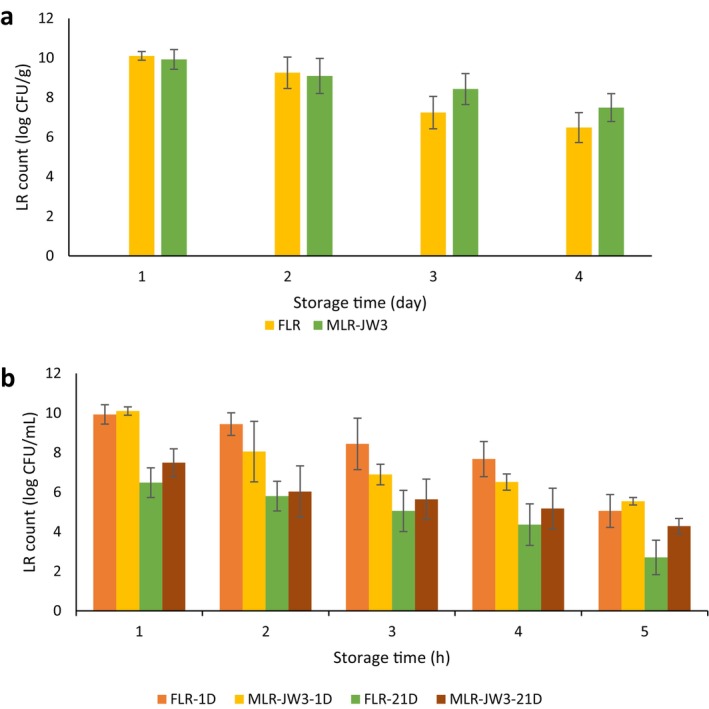
The viable count of free *Lacticaseibacillus rhamnosus* (FLR), microencapsulated *LR* with formulation JW3 (MLR‐JW3) containing a 60:40 ratio of Jujube mucilage (JM) and whey protein isolate (WPI); during storage in goat milk dessert (GMD). Microencapsulation was performed using a double‐layer extrusion method, with sodium alginate in the first layer and JM: WPI in the second layer. (a) Viable count during storage time and (b) viable count under simulated gastrointestinal conditions in the 1st and 14th of storage. Data (mean ± standard error) are from three replications.

Figure 5a shows how microcapsules affect the survival of LR at 4°C for 21 days, with assessments made every 7 days. On Day 1, the viable counts of FLR and MLR‐JW3 were 10.11 ± 0.22 and 9.93 ± 0.05 log CFU/g, respectively. After 21 days of storage, LRs lost 35.83% and 24.52% of their viability in FLR and MLR‐JW3, respectively, at 4°C. The final viable counts of FLR and MLR‐JW3 were 6.49 ± 0.76 and 7.50 ± 0.70 log CFU/g, respectively. Although both samples contained the required minimum count of probiotic bacteria in the GMD, the survival rate of MLR‐JW3 (75.48%) was greater than that of FLR (64.17%). In a similar study, Naseem et al. (2025) employed soy protein and Arabic gum as wall materials for the encapsulation of Lactobacillus strains and reported comparable outcomes. The observed effects can be attributed to strong electrostatic interactions and hydrogen bonding between soy protein and Arabic gum, which promote the formation of a denser and more cohesive matrix capable of effectively entrapping the encapsulated material. These results align well with our findings. After 21 days, we did not observe any noticeable reduction in the bacterial count across all the samples. The decrease in bacterial levels during storage is due mainly to the oxidation of lipids in the cell membrane. Therefore, it is important to consider the factors that affect how quickly lipid oxidation happens and how long it lasts, such as moisture levels and temperature, while storing the samples (Soltani Lak et al. 2021). In addition, low temperature was more favorable for maintaining the viability of the composite capsules during storage, because the metabolism of probiotic cells was much lower under low temperature, and the nutrients in the capsules were consumed slower. These properties led to the enhancement of the storage stability and prolongation of the shelf‐life of probiotic products (Li et al. 2019).

According to the research of Hoobin et al. (2013), adding inulin and WPI as a wall material significantly improved cell viability during storage. They also noted that molecular mobility and moisture content are key factors that affect cell survival throughout the storage period (Hoobin et al. 2013). Perez‐Gago and Krochta (2001) noted that carbohydrates might provide better protection for cells than WPI wall material. This advantage is due to their lower permeability to oxygen and moisture (Perez‐Gago and Krochta 2001). In related studies, *Lactobacillus plantarum* 21,805, when encapsulated with whey protein and dextran conjugates, showed only a slight reduction of 0.33 log CFU/mL after being stored for 90 days at 4°C (Guo et al. 2022).

### 3.11 Survival of FLR and MLR Exposed to SGC

On the basis of the data reported in Figure 5b, the survival rates of FLR and MLR‐JW3 were 53.50%, 68.82%, 46.55%, and 70.98%, respectively, on the 1st and 21st days of storage. In all samples, the number of viable bacteria decreased when the samples were exposed to acidic and bile conditions. However, the decrease was significantly less pronounced in the encapsulated samples. In all samples, the viability of MLR‐JW3 was greater than that of FLR, which revealed that the JM and WPI combination (0.6:0.4) was well protected against harmful SGC. When alginate is used as a barrier to encapsulate bacteria, it interacts with water to create hydrogels. These hydrogels offer some coverage for LRs, keeping them safe from gastrointestinal fluids and increasing their viability (Nazzaro et al. 2009). Despite being encapsulated, the bacteria in the gastrointestinal tract remain in close contact with acids and bile salts for a long time. As a result, the gastrointestinal fluid can seep into the alginate hydrogels that surround the probiotics, which helps them break free from their capsules. Unfortunately, this can negatively affect their viability (de Araújo Etchepare et al. 2016). Interestingly, when JM and WPI are incorporated within the framework of alginate‐based microcapsules, electrostatic interactions occur between JM + WPI and alginate. This interaction results in a sturdier capsule wall, which offers better protection against the invasion of gastrointestinal fluids. Similarly, combining proteins and carbohydrates as wall materials has been shown to increase the survival rate of encapsulated bacteria (Peredo et al. 2016). The results of similar studies also revealed a greater reduction in free bacteria than in microencapsulated bacteria under SGC. Nazzaro et al. (2009) reported improved acid and bile resistance in *L. acidophilus* when encapsulated within alginate‐inulin‐xanthan gum microcapsules. Specifically, the encapsulated cells exhibited only a 1 log CFU/mL reduction in viability after exposure to SGC, whereas a 5 log CFU/mL reduction was observed in free cells (Kim 2023). Similarly, *Lactobacillus* cells encapsulated in alginate beads significantly improved survival under SGC compared with free cells, with less than 1 log CFU/g loss in simulate gastric fluid (SGF) and a retention of ~6 log CFU/g after exposure to simulate intestinal fluid (SIF). Although the beads disintegrated during the intestinal and colonic phases, viable cells (4 log CFU/mL) were released into the SIF and remained stable. The released cells maintained functional properties similar to those of the original strain after 6 h of GI transit simulation (Zhao et al. 2012). The combination of whey protein and JM can improve bead survival during SGC. For example, the Maillard conjugate was formed from quinoa protein isolate and gum Arabic. This combination has proven to be quite effective for encapsulation, showing its ability to improve the bioaccessibility of the bioactive components that are encapsulated (Chen et al. 2023).

## 4 Conclusion

In this study, MLR was extensively assessed for its ability to assess cell viability under harsh conditions, such as heat and acid + salt. The findings demonstrated that the use of probiotic microencapsulation is feasible via a double‐layer extrusion process utilizing JM and WPI, which can increase the survival capacity of LR. The microcapsules produced exhibited an appropriate size and morphological structure conducive to microbial cell growth and survival. Among the various formulations for JM and WPI (60:40) ratios, JW3 was selected on the basis of its maximum EE% and survival ability under harsh conditions. Compared with free bacteria, MLR‐JW3 significantly improved the survival rate of cells in GMD during both the gastrointestinal transition and the storage period, especially. In the present investigations, texture and color parameters improved in MLR‐JW3 goat milk. Therefore, the use of the JM + WPI combination in the bead wall formulation provides a creative approach and new technique for protecting the LR during harsh conditions. Additionally, the results of this study point to an exciting potential for use in a variety of probiotic milk‐based desserts, particularly through the development of probiotic microcapsules. Future studies are recommended to investigate the long‐term stability and sensory acceptance of goat milk desserts containing multilayered microcapsules under various storage and distribution conditions. The optimization of different ratios of mucilage and protein‐based coatings, as well as the incorporation of other natural biopolymers, could further enhance encapsulation efficiency and probiotic viability. Additionally, exploring the release behavior of LR in real gastrointestinal models and expanding this encapsulation system to other probiotic strains and dairy or plant‐based desserts may provide valuable insights for functional food innovation and large‐scale production.

## Author Contributions

**Seyed Saeed Sekhavatizadeh:** writing – original draft and project administration. **Sara Baleshzar:** visualization.

## Funding

The authors have nothing to report.

## Ethics Statement

The authors have nothing to report.

## Conflicts of Interest

The authors declare no conflicts of interest.

## Supporting information


**Data S1:** fsn371450‐sup‐0001‐Supinfo.docx.

## Data Availability

The data that support the findings of this study are available from the corresponding author upon reasonable request.
