# Supplementary material for: Preparation and Characterization of Multilayered Microcapsules of Lacticaseibacillus rhamnosus Encapsulated With Sodium Alginate, Jujube Mucilage, and Whey Protein Isolate in Goat Milk Dessert
Source: Food Sci Nutr. 2026 Feb 12;14(2):e71450. doi: 10.1002/fsn3.71450 (PMC12895132; doi:10.1002/fsn3.71450)
Supplement: Supplementary file 1 — Data S1: fsn371450‐sup‐0001‐Supinfo.docx. [file FSN3-14-e71450-s001.docx]

Table 1s: The second layer component of MLR by extrusion

| WPI (%) | JM (%) | Treatment |
| --- | --- | --- |
| 0.8 | 0.2 | JW1 |
| 0.6 | 0.4 | JW2 |
| 0.4 | 0.6 | JW3 |
| 0.2 | 0.8 | JW4 |

jujube mucilage (JM); whey protein isolate (WPI)
